# The Influence of UV Radiation on the Degradation of Pharmaceutical Formulations Containing Quercetin

**DOI:** 10.3390/molecules25225454

**Published:** 2020-11-20

**Authors:** Iwona Golonka, Stanisław Wilk, Witold Musiał

**Affiliations:** Department of Physical Chemistry and Biophysics, Wroclaw Medical University, Borowska 211A, 50–556 Wroclaw, Poland; iwona.golonka@umed.wroc.pl (I.G.); stanislaw.wilk@gmail.com (S.W.)

**Keywords:** photosensitivity, flavonol, gel, quercetin-polymer interactions

## Abstract

The aim of this study was to assess the photostability of quercetin in the presence of anionic and nonionic polymeric gels with varied compositions of an added component—glycerol. The samples were irradiated continuously at constant temperature. The stability of quercetin in solution and incorporated into the gels was evaluated by an UV-Vis spectrophotometer. FTIR spectroscopy (Fourier-transform infrared spectroscopy) was used to detect the changes in the structure of quercetin depending on the polymer used in the gel, and on the exposure time. Photostabilization is an important aspect of quality assurance in photosensitive compounds. The decomposition rate of quercetin in the ionic preparation of polyacrylic acid (PAA) with glycerol was 1.952·10^−3^ min^−1^, whereas the absence of glycerol resulted in a decay rate of 5.032·10^−4^ min^−1^. The formulation containing non-ionic methylcellulose resulted in a decomposition rate of quercetin in the range of 1.679·10^−3^ min^−1^. The decay rate of quercetin under light influence depended on the composition of the gel. It was found that the cross-linked PAA stabilized quercetin and the addition of glycerol accelerated the photodegradation.

## 1. Introduction

Ultraviolet (UV) radiation is a part of the natural sunlight spectrum that reaches the Earth’s surface. The UV portion of the total sunlight does not exceed 5–6%, of which, under normal circumstances, more than 95% is UVA (320–400 nm) radiation and the rest is UVB (290–320 nm) light; UV-C radiation (200–280 nm) is actually completely absorbed by the atmosphere [1]. UVA impact is related to geography, altitude, season and day, weather conditions, and reflection. It penetrates deeper into the skin compared to UVB light, and may interact with both epidermal and skin cells [2]. Internal UVA solar irradiation accounts for approximately 25% (or 5–10 W/m^2^) of external irradiation and is approximately 60 times greater than irradiation with fluorescent light [3]. The stability of pharmaceutical gels varies depending on the gel type under consideration, and decomposition and dissolution may take place [4]. UV radiation and temperature are destructive factors enhancing the decomposition of pharmaceuticals, leading to toxic products [5].

Since the Sixties there has been a lot of interest in polymeric gels [6]. Due to their high water content, most hydrogel structures possess excellent biocompatibility. Polyacrylic acid (PAA) or methylcellulose (MC) are widely used by the pharmaceutical industry in gels applicable to many fields, including drugs and tissue engineering [7,8]. When formulated in an aqueous environment, PAA reacts with atmospheric oxygen. The consequence of this process is permanent reduction or loss of the gel’s viscosity. This process is catalyzed by sunlight, UV radiation, and the presence of certain metals in the solution [9]. The manufacturer states that the trace presence of iron ions and other transition metals in the gel may accelerate the PAA decomposition process [10]. According to the producer, PAA is not subject to aerobic degradation at pH values greater than 10; therefore, gel alkalization can prevent the loss of its rheological properties during irradiation. A similar effect of photodegradation inhibition can be obtained by adding water-soluble UV absorbers to the gel, e.g., benzophenone-2 [11] and benzophenone-4 (CTFA). In many gel formulations, these absorbers are used in combination with disodium or tetrasodium salts of ethylene diamine tetra-acetic acid (EDTA) [12,13]. The gamma-irradiated polyvinyl alcohol/MC blends possess a higher activation energy than the non-irradiated blends depending on the stage of thermal decomposition. This is due to the higher crosslink density of the polyvinyl alcohol component [14]. PAA is a polyelectrolyte capable of forming ions when subjected to an aqueous environment, causing considerable swelling due to hydrogen interactions or covalent bond formations. PAA is not toxic and is very sensitive to temperature and pH changes. Recent studies on swollen hydrogels based on PAA showed good mechanical and physical characteristics, such as elongation, strain, and tension resistance, among other properties. On the other hand, cellulose derivatives have high rigidity; some can swell and are soluble in water or other solvents depending on the extent of their degree of substitution. When combined with other materials, they can ensure biodegradability of the composite materials since they are polysaccharide derivatives. They can also enhance physical hydrogel properties.

Quercetin is an interesting compound due to its structure-related properties, and was investigated in the terms of the stability of pharmaceutical formulations, including chitosan nanoparticles [15], and emulsions with diversified lipid content [16]. In addition to health-promoting properties, it absorbs radiation and is therefore used in sunscreens. However, it is an unstable compound, and despite many studies, researchers do not have complete knowledge regarding the decay products of quercetin under the influence of various factors and the reactions they may cause. Quercetin decomposes if the pH value of the solution is higher than 10.2. The characteristic adsorption bands in the UV-Vis spectrum of quercetin in 50% (*v*/*v*) aqueous ethanol solution shift to higher wavelengths when the solution is adjusted to alkaline, and a new band appears at 321 nm [17].

Poor photostability is usually not an issue for polymeric gels. However, it may be important for active pharmaceutical ingredients applied on the skin, and exposed to the sunlight. Exposure to environmental factors, including UV-Vis radiation, may hinder the effective application of these preparations, and this research may provide more information on this topic. The purpose of this work was to analyze the effect of polymeric gel on the photostability of quercetin, applying the appropriate spectrophotometric methods.

## 2. Materials and Methods

### 2.1. Materials

The following reagents were used in the experimental part: quercetin (Quercetin ≥95% (HPLC), CAS 117-39-5, Sigma-Aldrich, Saint Louis, MO, USA), methylcellulose (MC) (CAS 9004-67-5, Sigma-Aldrich, Saint Louis, MO, USA), polyacrylic acid (Carbomer, CARBOPOL^®^ 980 NF POLYMER, CAS 9007-20-9, Lubrizol, Wickliffe, OH, USA), ethyl alcohol 96% (ethanol 96%, pure CAS 64-17-5, Chempur, Piekary Śląskie, Poland), sodium hydroxide (CAS 1310-73-2, Sigma-Aldrich, Saint Louis, MO, USA), and glycerol (CAS 56-81-5, Sigma-Aldrich, Saint Louis, MO, USA).

### 2.2. Methods

#### 2.2.1. The UV Irradiation

The samples were irradiated with a home-built system consisting of a high-pressure mercury lamp by HBO 200DC 200W (Osram, Munich, Germany) and a cuvette enabling safe irradiation. The measurements were carried out while a constant temperature of the irradiation system and the surrounding environment was maintained.

#### 2.2.2. Preparation

Briefly, 0.3% *w*/*w* gels were prepared using the following procedure. PAA was dispersed in distilled water in the volume required for the desired concentration of the gel. The mixture was stirred until thickening occurred and then neutralized by dropwise addition of 2.5 mmol NaOH, until a transparent gel formed. The quantity of NaOH was adjusted to achieve a gel with the desired pH value. During the preparation of the first gel, glycerol was added in appropriate proportions. A 4% gel containing MC in its composition was obtained by mixing the appropriate amount of MC with water at a suitable temperature. All gels were stabilized depending on the temperature for 24 h (Table 1).

#### 2.2.3. Calibration Curve

The gel components did not absorb light in the measured range of 210–440 nm, the recorded absorbance of quercetin in all samples did not exceed 1.5, and the obtained relationship between the absorbance and concentration was linear. The calibration curves of the standard substance were determined for the solution of quercetin in ethanol as well as for the 2A, 2B, and 2C preparations. The respective coefficients a and b of the linear equation y = ax + b describing the standard curve and the parameter errors were determined (Table 2). The coefficient of determination, normalized with values from zero to one, was determined by the R^2^ formula.

#### 2.2.4. FTIR Studies

FTIR measurements were performed using a Thermo Scientific Nicolet iS50 FT-IR Spectrometer with an attenuated total reflectance (ATR) device (Thermo Fisher Scientific, Waltham, MA, USA). The spectra of the preparations 2A, 2B, and 2C and their physical mixtures, as well as the individual components used in the present study, were recorded in the wavenumber range of 500–4000 cm^−1^.

#### 2.2.5. Spectrophotometric Method

The stability of preparations 2A, 2B, 2C, and QA was evaluated with a PG Instruments UV–Vis T60 spectrophotometer (Alab, Warszawa, Poland) interfaced with a computer. The samples were evaluated every 20 min from the initial stage, when the components were combined. The reported absorbance represents the average of five samples.

#### 2.2.6. Statistical Analysis

The experimental data were evaluated with Statistica 10.0 software (TIBCO Software, Palo Alto, CA, USA) using the Student’s t-test and F-test for linear correlation. The kinetic degradation rate constants were estimated with the Gauss–Newton algorithm according to the equation y = A_1_ + A_0_e^−kt^.

## 3. Results

### 3.1. Irradiated Substrates

In the initial experiment, the UVA and UVB spectra of the substrates were recorded every 20 min for a period of 220 min. During the whole irradiation process, no changes were observed in the spectra of the evaluated samples.

### 3.2. Quercetin Ethanolic Solution, QA

**Figure 1:**
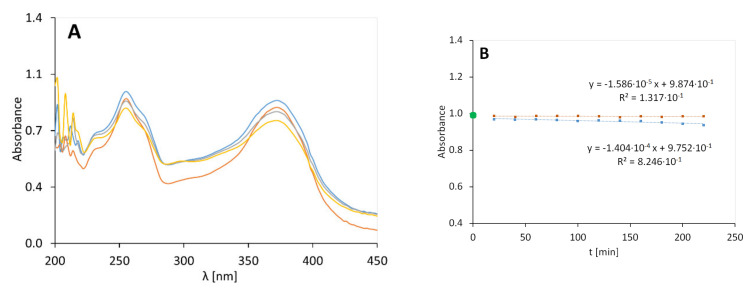
The graph on the left (**A**) shows the spectra of quercetin in ethanol solution (QA) exposed to radiation after 0 (**-**), 80 (**-**), 120 (**-**), and 200 (**-**) minutes. The graph on the right (**B**) shows the UV-induced quercetin ethanol solution degradation (orange dots are used for the results of the unexposed samples and blue dots are used for the result obtained for the irradiated samples).

The continuous irradiation of quercetin, in an ethanolic solution, resulted in its degradation, which increased with irradiation time—Figure 1.

After 200 min, some of the curves intersected, which may indicate the formation of an isosbestic point. The signal intensity at wavelengths of 250 nm and 375 nm decreased with increasing exposure time in a hypochromic effect. The kinetic plot, representing the absorbance decrease at 375 nm in time, is presented in Figure 1B. The slope of the absorbance plot over time for the exposed samples was fivefold higher compared to the unexposed samples; the rate constants of the first-order reaction were in the range of 1.404 × 10^−4^ min^−1^ and 1.586 × 10^−5^ min^−1^, respectively. The samples stabilized in the initial 20 min, and the initial absorbance in the spectra of the individual preparations oscillated around the estimated initial value of absorbance (green point—Figure 1B). The R^2^ for unexposed samples of quercetin in ethanol solution (QA) was 1.317 × 10^−1^, which indicated no linear relationship absorbance of the non-irradiated sample vs. time.

### 3.3. PAA Gel with Glycerol and Quercetin, 2A

**Figure 2:**
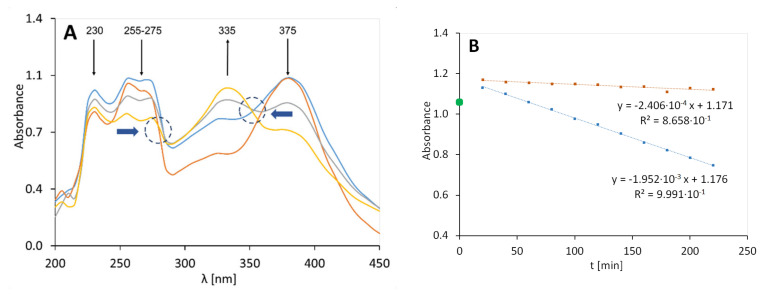
UV-Vis spectra of the polyacrylic acid (PAA) gel with quercetin and glycerol (preparation 2A), irradiated at 0 (**-**), 80 (**-**), 120 (**-**), and 200 (**-**) minutes (**A**). The graph on the right (**B**) shows the UV degradation of a gel containing PAA, quercetin, and glycerol (orange dots are used for the results of unexposed samples and blue dots are used for the result obtained for the irradiated samples).

The plots representing the absorption spectra of sample 2A after various exposure times are displayed in Figure 2. The arrows indicate the concentration change of the proposed evaluated substrates: a decrease in the intensity (↓) in the case of quercetin at ca. 255–275 nm and ca. 375 nm, and an increase in the intensity (↑) in the case of the degradation products at approximately 335 nm were observed (Figure 2); another band at 230 nm was also observed, which decreased, but it was not assigned to quercetin. In the case of preparation 2A, the signal at 230 nm was slight, compared to the signals observed in the spectra of the other preparations: 2B, 2C, and QA. During the first 20 min of irradiation the system was stabilized and the initial absorbance deviated from the further records (green point—Figure 2B).

The quercetin degraded tenfold faster in the irradiated gel than in the non-exposed preparation (Figure 2B). An increase in the signal at 335 nm was observed, which indicated the decomposition of quercetin. The decay constant of quercetin during irradiation, determined from the signal at 375 nm, was 1.952 × 10^−3^ min^−1^. The visible isosbestic point (blue arrows) at 285–290 nm suggests that at least one or more photoproducts form upon UV irradiation. The decrease in the signal intensity at 250 nm may be associated with the decay of the benzoyl II band occurring in the 240–280 nm range. Similarly, a decrease in the signal intensity at 375 nm may be associated with changes in the cinnamoyl band that appear at 320–385 nm.

### 3.4. PAA Gel with Quercetin, 2B

**Figure 3:**
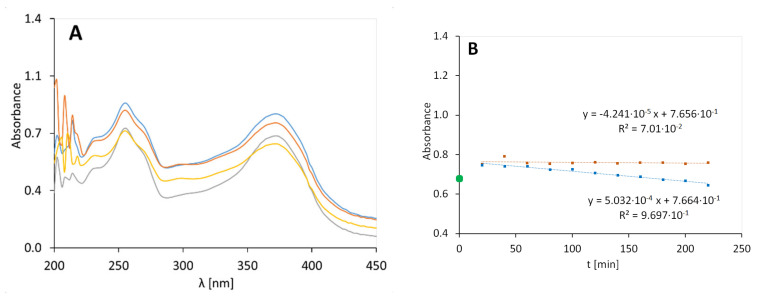
UV-Vis spectra of the PAA gel with quercetin (preparation 2B), irradiated at 0 (**-**), 80 (**-**), 120 (**-**), and 200 (**-**) minutes (**A**). The graph on the right (**B**) shows the UV degradation of a gel containing PAA and quercetin (orange dots are used for the results of unexposed samples and blue dots are used for the result obtained for the irradiated samples).

The UV-Vis spectra of PAA gel with quercetin (2B) are shown in Figure 3A. The rate constant of the irradiated preparation 2B was 5.032 × 10^−4^ min^−1^ and was close to the rate constant for the preparation containing MC (2C) when not exposed to radiation: 8.950 × 10^−4^ min^−1^. These observations support the concept of the stabilizing role of PAA in hydrogels containing quercetin.

### 3.5. MC Gel with Quercetin, 2C

**Figure 4:**
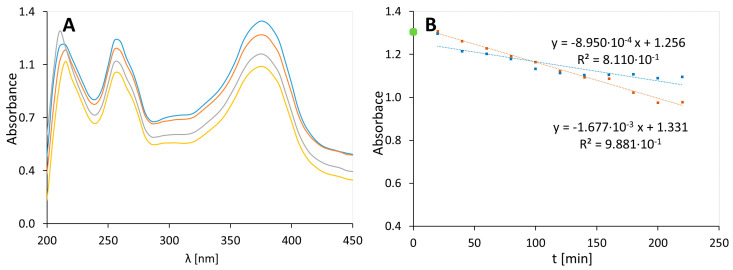
UV-Vis spectra of a methylcellulose (MC) gel with quercetin (preparation 2C), irradiated at 0 (**-**), 80 (**-**), 120 (**-**), and 200 (**-**) minutes (**A**). The graph on the right (**B**) shows the UV degradation of a gel containing a MC polymer and quercetin (orange dots are used for the results of the unexposed samples and blue dots are used for the result obtained of the irradiated samples).

The absorbance spectrum of a MC gel containing quercetin (2C) is shown on the left in Figure 4A.

As in the previous spectra, we observed signals about 220 nm, 250 nm, and 375 nm. In the spectrum of QA samples, a signal appeared at 220 nm. The intensity of these signals decreased with time in both the irradiated and non-irradiated samples. The kinetic plots shown in Figure 4B confirm the signal reduction at 375 nm in the spectrum of preparation 2C, both for the non-irradiated and irradiated samples. However, the reduction was two times higher for the irradiated samples compared to the non-irradiated samples. The rate constants were 1.677 × 10^−3^ min^−1^ and 8.950 × 10^−4^ min^−1^, respectively. This suggests the instability of quercetin in the gel based on MC, which increased in the presence of UV light.

### 3.6. Statistics

For a first-order reaction, as shown in the above figures, the plot of the absorbance [A] versus time is a straight line, where the slope of the straight line corresponds to the negative rate constant k. The unit of the rate constant of a first-order reaction is min^−1^. Thus, a change in the concentration does not change the numerical value of k in a first-order reaction. The linear correlation parameters of preparations 2A, 2B, 2C, and QA, which were stored in the dark and then exposed to radiation, are shown in Table 3. The highest rate constants among the irradiated samples were observed for gels based on PAA and glycerol, while the lowest rate constants were observed for quercetin dissolved in ethanol. Among the unexposed samples, the gels based on MC showed the highest rate constants, while the lowest rate constants were observed for quercetin dissolved in ethanol.

The linear correlation coefficient in Table 3 specifies the interrelationships between the selected variables, in our case, between absorbance and time of unexposed and exposed samples. To characterize the correlation of two features, two factors are considered: direction and strength. The results indicate that the correlation is positive, i.e., that an increase in the value of one feature is accompanied by an increase in the mean value of the second characteristic. In case of the unexposed preparations 2B and QA, the linear correlation coefficient is very low; that is, the linear relationship is not fulfilled, and thus, no changes in absorption are observed over time. The standard errors in estimating the coefficients a and b are less than 0.05. The same applies to the error of estimating y for all samples.

The purpose of using the F test for linear correlation was to determine whether the changes in the absorbance observed for an absorption time of 220 min in the unexposed and exposed conditions are statistically significant. The null hypothesis states that there is no difference between variables; instead, the relationship between the variables can be described by a linear function. A probability greater than 0.05 indicates that there is no reason to reject the null hypothesis. However, if the probability is lower than 0.05, the null hypothesis needs to be rejected, and an alternative hypothesis is needed. In the present study, significant changes occurred in the unexposed and irradiated 2A and 2C preparations and in the exposed 2B and QA preparations. The probability in these cases is lower than 0.05, which indicates that the absorbance changes over time. In the case of 2B and the unexposed QA preparation, the probability is greater than 0.05, and thus, there are no statistically significant changes.

### 3.7. Student’s t Test

The Student’s t test was used to test for statistically significant differences between the exposed and unexposed samples. The calculated parameters are shown in Table 4. Hypothesis H_0_ implies that the radiation does not affect the sample. The alternative hypothesis H_1_ implies that the radiation affects the sample.

With a confidence level of 5%, the t-test results rejected the null hypothesis and accepted the alternative hypothesis in all tested samples. In all cases, statistical differences of the behavior of quercetin in the different gels could be confirmed depending on whether the sample was irradiated or not.

### 3.8. FT-IR

**Figure 5:**
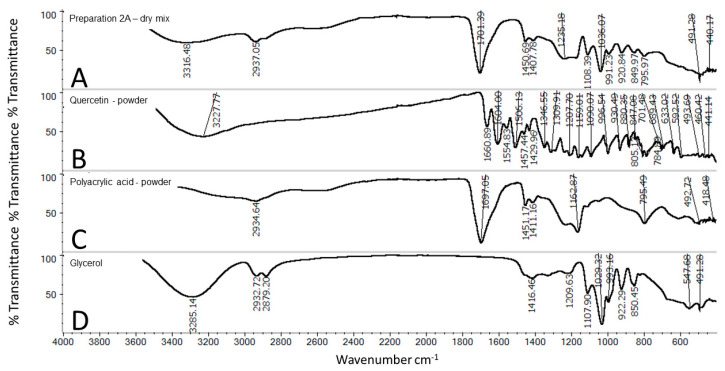
Fourier-transform infrared spectroscopy (FTIR) spectra of: physical mixture (**A**) of quercetin powder, PAA, glycerol in ratios reflecting the composition of 2A, quercetin (**B**), PAA (**C**) and glycerol (**D**).

Fourier-transform infrared spectroscopy (FTIR) spectra of: physical mixture of quercetin powder, PAA, glycerol in ratios reflecting the composition of 2A, quercetin, PAA and glycerol are presented in Figure 5. Within the glycerol spectrum there are several signals of specific functional groups: O-H stretching frequency was observed at 3285.14 cm^−1^, while C-H stretching was revealed via the peaks in the region of 2810–2950 cm^−1^. Bending of the C-O-H group was also observed in the region of 1400 to 1420 cm^−1^ of the C-O stretching of the primary alcohol, which was recorded at 1209.72 cm^−1^ [18] Comparing the spectrum of the mixture of the dry preparation 2A and the prepared gel not exposed to irradiation, we observed differences at 1701 cm^−1^, and 795 cm^−1^ derived from polyacrylic acid. The bands of C-H stretching in the region of 2810–2950 cm^−1^ should be ascribed to the presence of glycerol within the preparation. However, the signal at 548 cm^−1^ was not observable in the analyzed spectrum.

**Figure 6:**
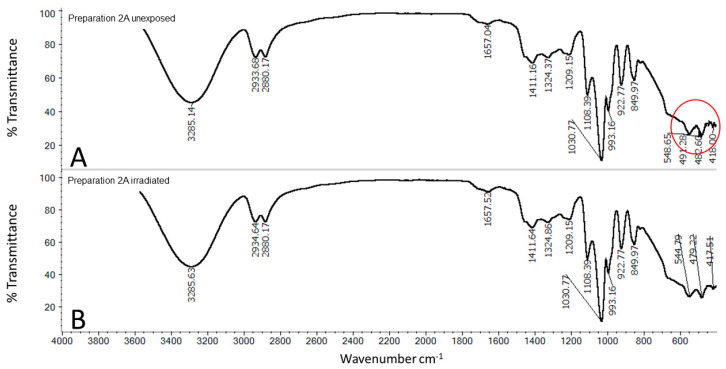
FTIR spectrum of freeze-dried preparation: (**A**) unexposed Preparation 2A, (**B**) irradiated Preparation 2A; composition of the Preparation 2A is given in the Table 1.

Figure 6 shows the spectra of the unexposed (Figure 6A) and irradiated (Figure 6B) preparation 2A. In both spectra of preparation 2A, in addition to the signals attributed to the polymer and quercetin, we observed signals from glycerol; the bimodal peak at approximately 2900 cm^−1^ can be attributed to CH stretching (2934 cm^−1^ and 2880 cm^−1^). The bending vibration of the CH_2_ group appears at approximately 1450 cm^−1^ as a shoulder of the 1411 cm^−1^ band. The 1411 and 993 cm^−1^ bands are attributed to the stretching of C-O in CH_2_OH, while the 1108 cm^−1^ band is attributed to the stretching of C-O in CHOH [19]. In the area close to 493 cm^−1^, we observed a band in the pure quercetin spectrum (Figure 5B), which disappeared in sample 2A at exactly 491 cm^−1^ (Figure 6B).

**Figure 7:**
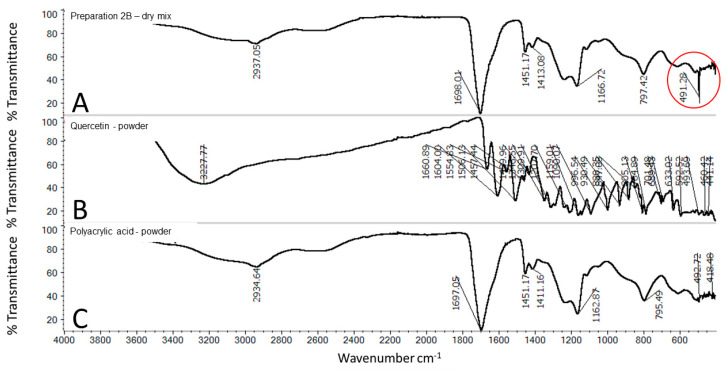
FTIR spectra of: physical mixture (**A**) of quercetin powder and PAA in ratios reflecting the composition of 2B, quercetin (**B**), and PAA (**C**).

The FTIR-ATR spectra of powdered quercetin and PAA are presented in Figure 7B,C, whereas the physical mixture of these components is presented in Figure 7A. OH group stretching was detected at 3227 cm^−1^, whereas OH bending of the phenol function was detected at 1346 cm^−1^. The C=O aryl ketonic stretching absorption was evident at 1661 cm^−1^. The C=C aromatic ring stretch bands were detected at 1603, 1554, and 1505 cm^−1^. The in-plane bending band of C-H in aromatic hydrocarbons was detected at 1309 cm^−1^, and out-of-plane bending bands were evident at 930, 817, 679, and 592 cm^−1^. The bands at 1207, 1159, and 1089 cm^−1^ were attributed to C-O stretching in the aryl ether ring, C-O stretching in phenol, and C-CO-C stretching and bending in ketone, respectively [20]. The typical structure of PAA exhibits a broad absorption band at 3056 cm^−1^ due to the -OH stretching vibration and a characteristic band at 1697 cm^−1^ attributed to C=O stretching. The absorption peak at 2934 cm^−1^ can be assigned to -CH stretching vibrations of the -CH_3_ and -CH_2_ functional groups. The band at 1200–1315 cm^−1^ is related to C-O stretching. The band at 1395–1450 cm^−1^ is assigned to C-O-H deformation vibration. The absorption band at 792 cm^−1^ is due to out-of-plane OH···O deformation, indicating the existence of strong interchain hydrogen bonds [21,22].

**Figure 8:**
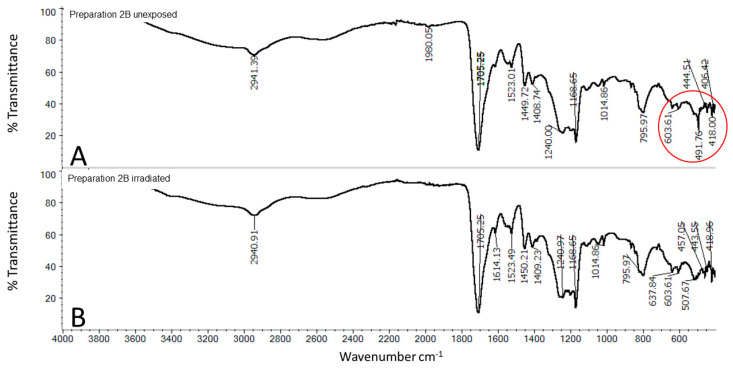
FTIR spectrum of freeze-dried preparation 2B—(**A**) unexposed Preparation 2B, (**B**) irradiated Preparation 2B; composition of the Preparation 2B is given in the Table 1.

The FTIR-ATR spectrum of the exposed PAA gel containing quercetin (Figure 8B) and stored in the dark (Figure 8A) presents some differences. Aromatic ring stretching bands of C=C were detected at 1614, 1550, and 1523 cm^−1^. The bands in the spectrum of the unexposed sample at wavenumbers 795, 1014, 1168, 1240, 1408, and 1449 were attributed to PAA, while 491, 603, 795, 1014, 1168, 1408, and 1523 were attributed to quercetin. Some signals overlapped. Comparing the spectrum of the preparation stored in the dark (2A) to the irradiated (2B), the spectra showed high variability in the fingerprint range of quercetin. In the case of irradiated 2B, the band at 491 cm^−1^, close to that revealed in pure quercetin (493 cm^−1^), disappeared and appeared at 507 cm^−1^; only slight signal shift occurred. The spectrum in the range of 1523 cm^−1^ to 1614 cm^−1^ attributed to C=C binding of the tensile vibration in the case of an irradiated preparation showed additional signals, which appeared as sharp peaks.

**Figure 9:**
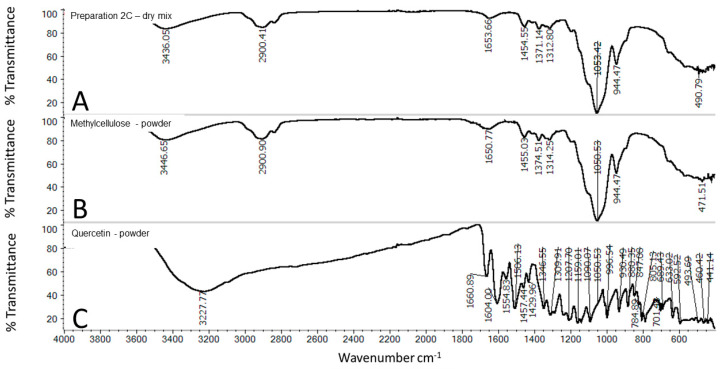
FTIR spectra of: physical mixture (**A**) of quercetin powder and MC in ratios reflecting the composition of 2C, MC (**B**), and quercetin (**C**).

According to Figure 9 the non-doped MC revealed an absorption band at 3446 cm^−1^, related to the stretching vibration ν of the hydroxyl group (O-H). The bands at 2900 were assigned to C-H stretching of the methyl groups of the MC [23]. The peaks at 1374 and 1452 cm^−1^ represented the vibration of deformation in the plane of δ (C-H). The absorption band at 1050 cm^−1^ was attributed to the C-O-C stretching mode of glycosidic units [24]. The peak at 944 cm^−1^ was related to methoxy groups.

**Figure 10:**
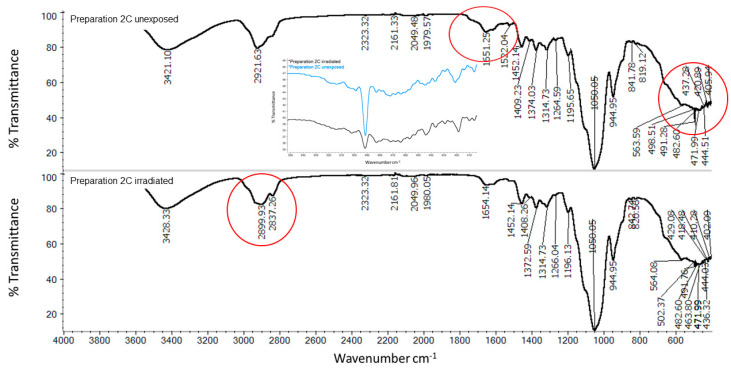
FTIR spectrum of freeze-dried preparation 2C—(**A**) unexposed Preparation 2C, (**B**) irradiated Preparation 2C; composition of the Preparation 2C is given in the Table 1. The inset shows the enlarged signal in the spectrum at approximately 491 nm, for unexposed preparation of 2C.

Differences in FTIR spectra between non-irradiated and irradiated samples 2C (Figure 10) could be observed in the C-H bond stretching range. In the second case (Figure 10B), there was a clear signal at 2837 cm^−1^ from the symmetric stretching vibration of C-H. In non-irradiated samples, we observed the C=C stretching of the aromatic ring signal at 1522 cm^−1^, which was no longer visible in the spectrum for irradiated samples. The signal from C=C binding for alkenes at 1651 cm^1^ was shifted to 1654 cm^−1^. In addition, in the exposed sample, we no longer observed a strong quercetin signal around 491 cm^−1^, close to the 493 cm^−1^ in the pure quercetin spectrum.

## 4. Discussion

The flavonoid compounds consist of a 15-carbon skeleton with two aromatic rings (A and B) connected to three carbon atoms, with oxygen atoms encapsulated in a heterocyclic C-ring [25,26]. The catechol structure of quercetin, i.e., the o-dihydroxy group in the B ring, is a potential radical target. The double bond between positions 2 and 3 of the C-ring, conjugated with the keto group in position 4, has the capacity to delocalize the uncoupled electron of the flavonoid radical. The C-3, C-5, and C-7 hydroxyl groups of the C and A rings are potential free-radical scavengers [27].

**Figure 11:**
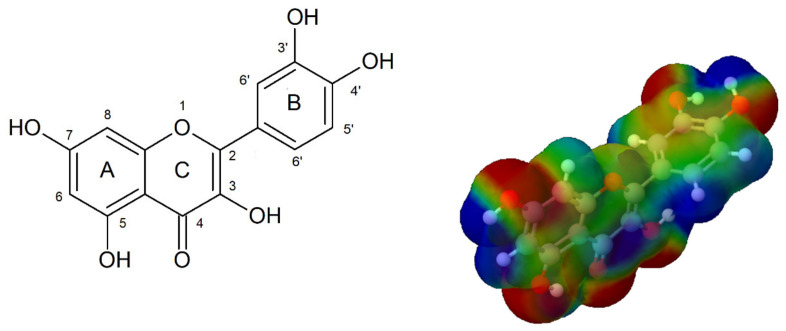
Quercetin structure and its molecular electrostatic potential (MEP) [28].

The three-dimensional model of the electrostatic potential map of quercetin is shown in Figure 11 on the right, where the semi-spherical blue shapes represent hydrogen atoms, whereas other spherical protrusions reflect individual atoms. The red areas of low potential are characterized by an abundance of electrons, and the blue areas of high potential are characterized by a relative absence of electrons. The blue positive region is prone to nucleophilic attacks, whereas the red negative region is sensitive to electrophilic attacks.

**Figure 12:**
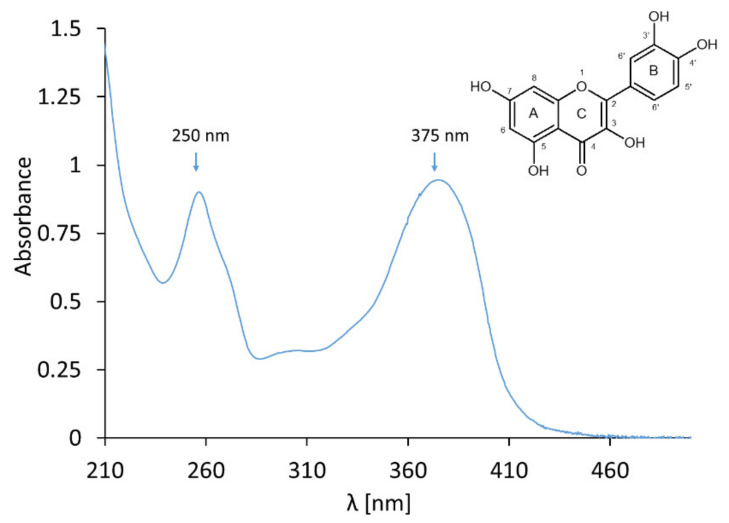
UV-Vis spectra of the ethanolic solution of quercetin.

However, using UV-Vis spectrophotometry, we observed that the quercetin ethanol solution absorbs UV radiation with a maximum absorbance in the UVA range at A_max_ at 375 nm, which is attributed to the B-ring, and in the UV-C range at A_max_ 258 nm, which is attributed to the A–C benzoyl system. A weak band with an absorption maximum of approximately 300 nm attributed to the C-ring only was also detected (Figure 12) [27,29,30]. Depending on the composition of the preparation and the exposure time, the absorbance of individual signals changes, which is related to the structure of quercetin. Under the influence of irradiation of PAA and glycerol-based gels containing quercetin (preparation 2A) and gels with quercetin based on MC (preparation 2C), we observed a time-dependent decrease in the absorbance signal at 375 nm on the UV-Vis spectrum. The rate of the decrease of absorption of this signal, over time of exposure, is one order of magnitude higher than that of the decrease of this signal in the spectra of the gels based on PAA alone or the ethanolic solution of quercetin. From these data, it can be concluded that the gel based on PAA stabilizes quercetin most efficiently. In the case of preparation 2A, a decrease in the absorbance at 250 nm and 230 nm and the appearance of the signal at 335 nm was also observed. The maximum at 375 nm underwent a gradual blueshift to 335 nm, indicating a loss of conjugation of the chromophore. The presence of at least two isosbestic points suggests that at least one or more photoproducts formed upon UV light exposure. In the case of an unexposed sample, we did not observe a signal at 335 nm or an isosbestic point. This may indicate that light was the only catalyst for the entire degradation process of the irradiated preparation 2A. There is a possibility that the degradation products of quercetin react with glycerol. Chain reactions were discussed by Chaaban et al. [31]. Only few studies can be found in the literature that report the effect of light on quercetin, and flavonoids in general, at the molecular level, and some of the reports are contradictory. Quercetin has been proven to be photostable in propylene glycol solutions [32]; its photodegradation has been observed in creams, unless protected by lipid micelles [33] or cellulose [34]. 

An interesting result was observed in the gel based on MC (preparation 2C), where despite the lack of light, the signal absorbance at 375 nm attributed to quercetin decreased. This indicates the instability of the compound in the gel based on MC, and the reaction of quercetin with MC despite the lack of light. The results in this study were confirmed by appropriate statistical analyses.

From the FTIR-ATR spectra, the fewest changes during irradiation were observed in the 2B formulation. These were changes in the assessed range at near to 490 cm^−1^ attributed to quercetin. In the case of preparation 2C, many differences were observed in the FTIR spectra of the irradiated and the unexposed samples. The bands at 2900 cm^−1^ assigned to C-H stretching, due to the presence of the CH_3_ group of MC, were shifted to 2921 cm^−1^ and separated by two signals, 2899 cm^−1^ and 2837 cm^−1^, in the irradiated formulation. This may indicate a reaction between the polymer and quercetin. In preparation 2A, we observed changes in the spectra of irradiated and nonradiated samples.

In the case of an ethanolic solution of quercetin, different researchers induced the degradation of quercetin by both UVA and UVB light, yielding a single product derived from oxidation and addition of an ethanol molecule to the 2,3-double bond. The same mechanism occurred when quercetin was dissolved in alkaline solutions [35]. Quercetin absorbs UV radiation, suggesting that one plausible photoprotective mechanism would be direct absorption of UV radiation, thereby preventing the formation of ROS and direct DNA damage [30,36,37]. However, other photodegradation products are 2,4,6-trihydroxybenzaldehyde, 2-(3′,4′-dihydroxybenzoyloxy)-4,6-dihydroxybenzoic acid, and 3,4-dihydroxyphenyl-ethanol [27]. Flavonoids are photoprotective as a result of their UV absorption, and their ability to act as direct and indirect antioxidants, and anti-inflammatory and immunomodulatory agents. Thus, they provide exciting platforms for the development of photoprotective materials. When used in combination with titanium dioxide, the so-called sun protection factor (SPF) is approximately 30 and therefore increases photoprotective properties [38]. Quercetin is unstable when exposed to atmospheric oxygen, which results in its degradation and complicates analytical characterization. The oxidative degradation involves several subsequent chemical reactions such as hydroxylation or dimerization. As the oxidation continues, a loss of isosbestic points indicates the decomposition of the oxidation product. The increase in the absorption band at 293 nm supports the formation of 3,4-dihydroxybenzoic acid (3,4-DHBA) [39]. Some factors may stabilize quercetin, e.g., the incorporation of quercetin into lipophilic particles, considered as an effective strategy for increasing its stability in dermatological products [33]. In addition, glycerol could enhance the oxidative stability of quercetin, according to Jerzykiewicz et al. [40]. However, the results suggest that glycerol is a decomposing factor for the stability of quercetin.

## 5. Conclusions

Photostability data, among other stability-indicating data, are crucial for the development of pharmaceutical formulations; hence, the industrial requirements of complete stability studies for most pharmaceutical formulations and drug substances are a priority. We can conclude that there was only a minor effect of oxygen on the stability of quercetin-containing gels, but that there was a large effect of light on the gel stability. The gel composition is also important. Our research shows that glycerol and MC accelerated the degradation of quercetin.

## Figures and Tables

**Table 1:** Preparation of Polymeric Gels.

Sample Acronym	PA [g]	MC [g]	NaOH [g]	Glycerol [g]	Aqua [g]	QA ** [mg]
Gel 1A	0.3	-	q.s. *	25.0	73.7	-
Gel 1B	0.3	-	q.s. *	-	94.7	-
Gel 1C	-	4	-	-	96.0	-
Preparation 2A	0.3	-	q.s. *	25.0	73.7	0.2
Preparation 2B	0.3	-	q.s. *	-	94.7	0.2
Preparation 2C	-	4	-	-	96.0	0.2

* 2.5 mmol of NaOH solution was added to the polymer dispersion, ** in ethanolic quercetin solution.

**Table 2:** Linear Correlation Parameters for the Standard Curve y = ax + b.

Preparation	2A *	2B *	2C *	QA **
slope factor a	559.36	833.30	596.80	24406
standard error for a	19.48	115.14	85.63	368.1
coefficient b	0.092	0.091	0.333	−0.017
standard error for b	0.028	0.111	0.094	0.010
linear correlation coefficient	0.994	0.929	0.924	0.998

Preparations 2A, 2B, 2C—polymeric preparations of quercetin described in the text, QA—ethanolic solution of quercetin, *—concentration in *w*/*w*%, **—concentration in mol/l.

**Table 3:** Linear Correlation Parameters for the Obtained Data.

Parameter	Preparation 2A		Preparation 2B		Preparation 2C		QA ***	
	Irradiated	Unexposed	Irradiated	Unexposed	Irradiated	Unexposed	Irradiated	Unexposed
slope factor a	−1.952·10^−3^	−2.406·10^−4^	−5.03210^−4^	−4.241·10^−5^	−1.679·10^−3^	−8.950·10^−4^	−1.404·10^−4^	−1.586·10^−5^
coefficient b	1.176	1.171	7.664·10^−1^	7.656·10^−1^	1.331	1.256	9.752·10^−1^	9.874·10^−1^
standard error a	1.974·10^−5^	3.157·10^−5^	2.966·10^−5^	5.149·10^−5^	6.138·10^−5^	1.440·10^−4^	2.159·10^−5^	1.358·10^−5^
standard error b	2.678·10^−3^	4.282·10^−3^	4.023·10^−3^	6.985·10^−3^	8.326·10^−3^	1.954·10^−2^	2.928·10^−3^	1.842·10^−3^
linear correlation coefficient	9.991·10^−1^	8.658·10^−1^	9.697·10^−1^	7.009·10^−2^	9.881·10^−1^	8.110·10^−1^	8.246·10^−1^	1.317·10^−1^
standard error of y estimation	4.141·10^−3^	6.622·10^−3^	6.221·10^−3^	1.080·10^−2^	1.287·10^−2^	3.022·10^−2^	4.528·10^−3^	2.848·10^−3^
parameter F	9.783·10^3^	5.808·10^1^	2.878·10^2^	6.783·10^−1^	7.482·10^2^	3.860·10^1^	4.232·10^1^	1.365·10^0^
p	<0.05	<0.05	<0.05	4.314·10^−1^	<0.05	<0.05	<0.05	2.727·10^−1^

*** quercetin in ethanol solution (QA) C = 3970·10^−5^ mol/dm^3^.

**Table 4:** Parameters of the Student’s *t* Test.

Parameter	Preparation 2A	Preparation 2B	Preparation 2C	QA ***
t	4.598·10^1^	7.754·10^0^	1.072·10^1^	5.506·10^0^
p	<0.0001	<0.0001	<0.0001	3.770·10^−4^

*** quercetin in ethanol solution (QA) C = 3970·10^−5^ mol/dm^3^.
